# A Novel PL9 Pectate Lyase from *Paenibacillus polymyxa* KF-1: Cloning, Expression, and Its Application in Pectin Degradation

**DOI:** 10.3390/ijms20123060

**Published:** 2019-06-22

**Authors:** Ye Yuan, Xin-Yu Zhang, Yan Zhao, Han Zhang, Yi-Fa Zhou, Juan Gao

**Affiliations:** 1 School of Life Sciences, Northeast Normal University, Changchun 130024, China; yuany268@nenu.edu.cn (Y.Y.); zhangh800@nenu.edu.cn (H.Z.); zhouyf383@nenu.edu.cn (Y.-F.Z.); 2 School of Biological Science and Technology, University of Jinan, Jinan 250022, China; zhangxinyu950512@163.com (X.-Y.Z.); zhaoyan_1994@126.com (Y.Z.)

**Keywords:** pectate lyase, *Paenibacillus polymyxa*, pectins, degradation, *Lactobacillus*

## Abstract

Pectate lyases play an important role in pectin degradation, and therefore are highly useful in the food and textile industries. Here, we report on the cloning of an alkaline pectate lyase gene (*pppel9a*) from *Paenibacillus polymyxa* KF-1. The full-length gene (1350 bp) encodes for a 449-residue protein that belongs to the polysaccharide lyase family 9 (PL9). Recombinant PpPel9a produced in *Escherichia coli* was purified to electrophoretic homogeneity in a single step using Ni^2+^-NTA affinity chromatography. The enzyme activity of PpPel9a (apparent molecular weight of 45.3 kDa) was found to be optimal at pH 10.0 and 40 °C, with substrate preference for homogalacturonan type (HG) pectins vis-à-vis rhamnogalacturonan-I (RG-I) type pectins. Using HG-type pectins as substrate, PpPel9a showed greater activity with de-esterified HGs. In addition, PpPel9a was active against water-soluble pectins isolated from different plants. Using this lyase, we degraded citrus pectin, purified fractions using Diethylaminoethyl (DEAE)-sepharose column chromatography, and characterized the main fraction MCP-0.3. High-performance gel permeation chromatography (HPGPC) analysis showed that the molecular mass of citrus pectin (~230.2 kDa) was reduced to ~24 kDa upon degradation. Ultra-performance liquid chromatography - tandem mass spectrometer (UPLC-MS) and monosaccharide composition analyses demonstrated that PpPel9a worked as an endo-pectate lyase, which acted primarily on the HG domain of citrus pectin. In vitro testing showed that the degradation product MCP-0.3 significantly promotes the growth of *Lactobacillus plantarum* and *L. rhamnosus*. In this regard, the enzyme has potential in the preparation of pharmacologically active pectin products.

## 1. Introduction

Found widely in plants, pectins are composed of d-galacturonic acid (GalA), l-rhamnose (l-Rha), d-galactose (d-Gal), and l-arabinose (l-Ara) [1,2]. They consist of three structural domains: 1) homogalacturonan (HG) that is mainly composed of α-1,4-linked d-GalA and thought of as “smooth regions” in pectin, 2) rhamnogalacturonan I (RGI) domains, and 3) rhamnogalacturonan II (RGII) domains that are both rich in neutral saccharides and thought as the “hairy regions” [3]. The acidic residues in pectin are usually esterified or acetylated, which makes pectin a diverse family of anionic structural hetero-polysaccharides. Pectin shows great potential in the food, pharmaceutical and polymer industries[1,4,5]. Recent research has shown that pectin has many biological activities, such as anti-tumor and antioxidation activities, as well as immune regulation activity [4,6]. However, the molecular weight of natural pectin is too large to be absorbed and utilized by the body. Modified pectin with lower molecular weight fragments and lower degrees of esterification would be more favorable for absorption and utilization, and potentially exhibit greater biological activity. Previous studies reported that low-molecular-weight modified citrus pectin (MCP) had greater activity against colon, breast, and gastrointestinal cancer, likely due to decreased expression of galectin-3 [4,7,8]. Low-molecular-weight MCP (i.e., 3 kDa to 30 kDa) exhibited greater anti-cancer activity by inhibiting the migration, aggregation, and proliferation of cancer cells compared to parent MCP with molecular weights greater than 30 kDa [9]. Low-molecular-weight MCP also promoted the growth of *Bifidobacterium longum*, suggesting that it has potential as a prebiotic agent [10].

Pectin degradation can be achieved by temperature, pH, or enzymatic modification [11]. Among these, enzymatic modification has a significant advantage [12,13]. Pectinolytic enzymes, known as pectinases, are a group of enzymes that include hydrolases, lyases, and oxidases, which play important roles in the degradation and modification of pectin [12,14]. Pectate lyase (EC 4.2.2.2), a member of the pectinase family, catalyzes the hydrolysis of α-1,4-glycosidic bonds to produce oligosaccharides with 4-deoxy-α-d-mann-4-enuronosyl groups at their non-reducing end [15,16]. Pectate lyase can function in either an endo- or exo-mode of action. Unmethylated pectins or pectins with a low degree of methylation are the preferred substrates for pectate lyases [17]. Depending on their amino acid sequence, pectate lyases (PL) are divided into five families: PL1, 2, 3, 9, and 10 [18]. 

These enzymes are widely distributed in microorganisms and plants. Previously, pectate lyases cloned from bacteria and fungi, such as *Bacillus*, *Erwinia*, *Aspergillus*, *Clostridium*, *Paenibacillus*, *Streptomyces*, and *Klebsiella* sp., have been reported [16,17,19]. Various industrial applications with these enzymes have been investigated, including fruit juice/wine clarification, plant fiber processing, and paper production [19,20,21]. Pectinases are good tools in pectin degradation and preparation of bioactive oligosaccharides [22]. However, there are few reports on the use of pectate lyase in pectin degradation or pectin structure analysis. In the present study, we cloned a pectate lyase gene belonging to PL9 from *Paenibacillus polymyxa* KF-1 and determined the substrate specificity of the recombinant pectate lyase PpPel9a. The degradation product of citrus pectin by PpPel9a was prepared, analyzed, and its prebiotic activity was evaluated.

## 2. Results and Discussion

### 2.1. Gene Cloning and Sequence Analysis of PL9 Pectate Lyase from *P. polymyxa* KF-1

Previously, four pectate lyases were identified from the fermentation broth of *P. polymyxa* KF-1 by liquid chromatograph-mass spectrometer/mass spectrometer (LC-MS/MS) analysis. These enzymes belong to PL families 1, 3, 9, and 10 [23]. In our previous work, a pectate lyase named PpPel10a was cloned from *P. polymyxa* KF-1 and was identified to be a member of PL10 family [23]. Here, the protein with the UniProt accession number E3E7F9 (NCBI protein ID MK809514) was encoded by an open reading frame (ORF) of 1350 bp. The protein of 449 amino acids was named as PpPel9a, and the N-terminal 34-amino acids was predicted to be the signal peptide sequence by signalP 5.0 server, which suggested the extracellular location of the protein. The *p*I value of PpPel9a without the signal peptide was predicted to be pH 5.07, similar to that reported for PL9 pectate lyase Pel-15H from *Bacillus* sp. strain KSM-P15 (4.60) [24], but lower than other PL9 enzymes, such as PelX from *Erwinia chrysanthemi* 3937 (7.7) [25], and Exo-PL from *E. chrysanthemi* EC16 (8.6) [26]. Analysis of the amino acid sequence by Uniprot confirmed that PpPel9a was a member of PL family 9 pectate lyase family, and the Pfam analysis indicated that PpPel9a had a β-helix superfamily domain. Multiple sequence alignment analysis showed that the amino acid sequence of PpPel9a has moderate similarity to other PL9 enzymes, such as pectate lyase Pel9A from *E. chrysanthemi* (1RU4) [27], rPel9A from *Clostridium stercorarium* F-9 (AB106865) [17], and rhamnogalacturonan lyase from *Bacteroides thetaiotaomicron* VPI-5482 (PDB accession no. 5OLQ) [28]. The sequence identities were 39.34%, 37.57%, and 42.53%, respectively. The low homology between PpPel9a and the reported PL9 enzymes indicates that PpPel9a may be a novel enzyme. So far, only several pectate lyases belonging to PL family 9 have been characterized, including Pel-15H from *Bacillus* sp. strain KSM-P15 (AB028878) [24], rhamnogalacturonan lyase BT4170 from *B. thetaiotaomicron* VPI-5482 (5OLQ) [28], Exo-PL from *E. chrysanthemi* EC16 (AAA24850) [26], PelL1 from *E. chrysanthemi* PY35 (AAF05308) [29], and PelX from *E. chrysanthemi* 3937 (CAB39324) [25]. The substrate specificity and biochemical properties of PL9 enzymes have yet to be clarified. In addition, the use of PL9 enzymes in the degradation of pectins had not been explored. Therefore, in the present study, we chose the gene encoding for the PL family 9 enzyme from *P. polymyxa* KF-1 for cloning, expression, and characterization.

**Figure 1:**
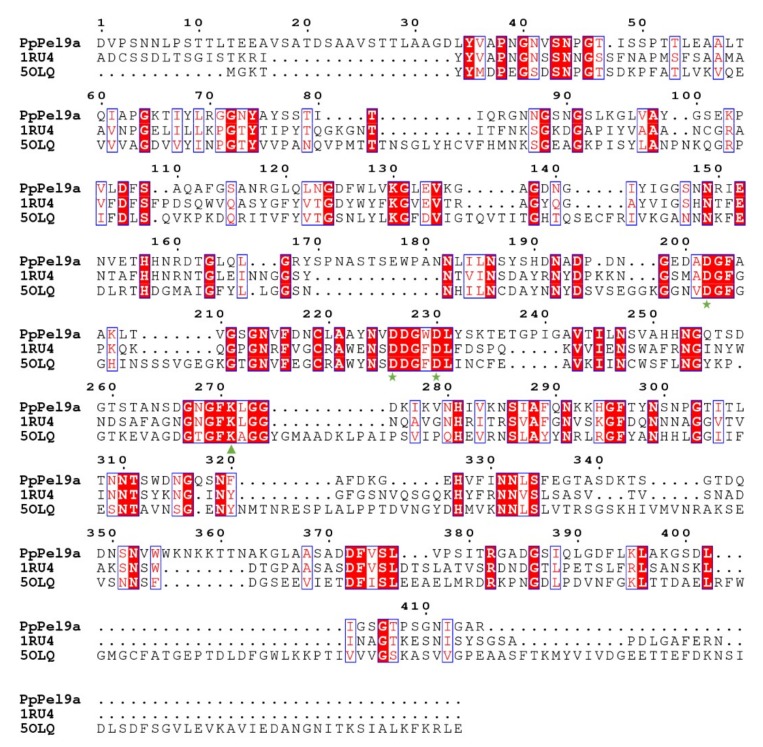
Alignment of amino acid sequence of PpPel9a with the PL9 family pectate lyases: Pel9A from *E. chrysanthemi* (1RU4) and rhamnogalacturonan lyase from *B. thetaiotaomicron* VPI-5482 (PDB accession no. 5OLQ). The conserved Asp were labeled by asterisk and the catalytic base was labeled by triangle.

So far, only two crystal structures have been reported for enzymes in the PL9 family (http://www.cazy.org/PL9_structure.html), namely rhamnogalacturonan lyase from *B. thetaiotaomicron* VPI-5482 (PDB accession no. 5OLQ) [28] and Pel9A from *E. chrysanthemi* (PDB accession no. 1RU4) [27]. Both lyases have a right-handed parallel β-helix fold with three short parallel β-sheets (PB1, PB2, and PB3) and ten turns, with two calcium ion binding sites (Ca-1 and Ca-2). The key components of the active site comprise Ca-1 on coils 5 and 6, and a lysine in coil 7 that functions as a catalytic base to abstract a proton from C5. The three-dimensional structure prediction by SWISS-MODEL indicates that PpPel9a may comprise a 10-coil parallel β-helix domain (Figure S1), similar to Pel9A from *E. chrysanthemi* (1RU4) [27]. ClustalW alignment of PpPel9a from *P. polymyxa* with the two characterized PL9 pectate lyase amino acid sequences highlighted three highly conserved calcium-binding sites: Asp202, Asp226, and Asp230 (Figure 1). Similar to the reported PL9 enzymes, the catalytic base in PpPel9a was predicted to be Lys271, which is different from that in PL1 pectate lyases where the catalytic base is an arginine (Arg198 in PL1 enzyme BsPelA from *Bacillus* sp. N16-5 [30], Arg240 in EcPelE from *E. chrysanthemi* [31], and Arg300 in BsPel from *B. subtilis)* [32]. In addition, PpPel9a and PpPel10a have different structures. PpPel9a has a right-handed parallel β-helix fold, whereas PpPel10a is predominantly α-helical with short β-strands and irregular coils [23].

### 2.2. Expression of Recombinant PpPel9a

After induction at 25 °C for 20 h, pectate lyase activity in the cytoplasm with polygalacturonic acid (PGA) as substrate, reached its maximum level (117.4 ± 4.82 U/mg). The recombinant enzyme was purified by Ni-NTA affinity chromatography, and the purified enzyme showed an approximate 4.5-fold increase in purity, and a recovery of 28.9% relative to the crude enzyme. Purification yielded 212 mg of enzyme/liter. This relatively high yield makes PpPel9a a good candidate for industrial applications. 

**Figure 2:**
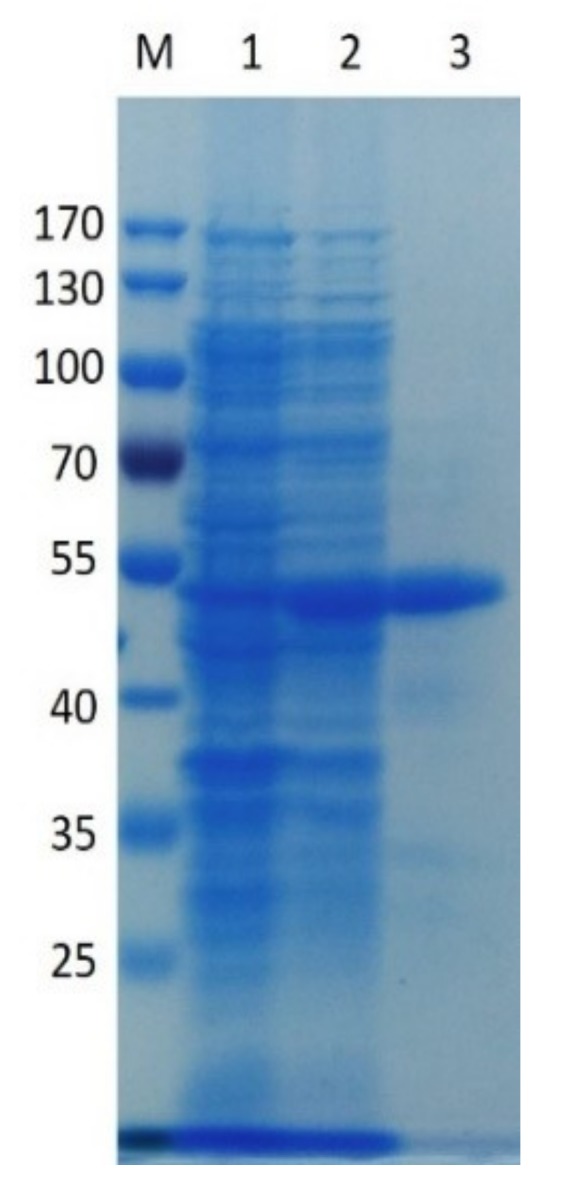
Molecular weight of PpPel9a determined by SDS-PAGE: (M) protein molecular weight markers (PageRuler Prestained Protein Ladder, Thermo Scientific) (1) supernatant of cell lysis from *E. coli* BL21 (DE3) cells; (2), supernatant of cell lysis from recombinant *E. coli* BL21 (DE3) cells harboring pET-28a-pppel9a plasmid; (3) recombinant enzyme PpPel9a purified from Ni-NTA agarose column.

Sodium dodecyl sulphate-polyacrylamide gel electrophoresis (SDS-PAGE) indicates that purified recombinant PpPel9a has a Mw of ~45.3 kDa, consistent with the predicted Mw (Figure 2). The Mw of PpPel9a was similar to that reported for PL9 enzyme PelL1 from *E. chrysanthemi* PY35 (43 kDa) [29], but lower than that reported for other PL9 pectate lyases, such as Pel-15H from *Bacillus* sp. strain KSM-P15 (69.55 kDa) [24], Exo-PL from *E. chrysanthemi* EC16 (76 kDa) [26], PelX from *E. chrysanthemi* 3937 (76.938 kDa) [25], and Pel9A from *C. stercorarium* F-9 (135.171 kDa) [17].

The specific activity of purified PpPel9a with PGA as substrate, was 298.5 ± 3.6 U/mg, higher than that reported for PL9 enzymes rPel9A from *C. stercorarium* (58 U/mg) [17] and Pel-15H from *Bacillus* sp. strain KSM-P15 (10.6 U/mg) [24], but lower than the PL1 pectate lyases, such as BacPelA from *B. clausii* (675.5 U/mg)[19] and Apel from *B. subtilis* (1010.0 U/mg) [33]. PL9 enzymes show lower activity against PGA than PL1 enzymes. This is likely due to the Lys catalytic base in PL9 enzymes being less potent than Arg in PL1 enzymes. In addition, the third calcium is important in increasing the acidity of the C5 proton that may be absent in PL9 enzymes [27]. However, PpPel9a exhibited higher pectate lyase activity than that of PpPel10a when using PGA and citrus pectin (CP) as substrates [23]. The specific activities with PGA using PpPel9a and PpPel10a were 298.5 ± 3.6, and 289 ± 4.9 U/mg, respectively, while the specific activities on CP by PpPel9a and PpPel10a were 107 ± 0.2, and 98.0 ± 3.6 U/mg, respectively.

### 2.3. Biochemical Characterization of PpPel9a

**Figure 3:**
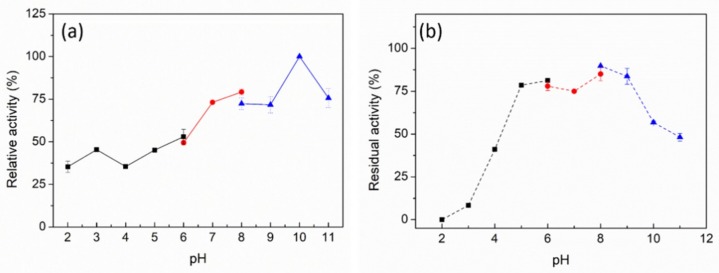
Effect of pH on activity (**a**) and stability (**b**) of PpPel9a. The enzyme activity was detected by using PGA as a substrate (0.2% *w*/*v*). The following buffers were used: 50 mM acetate buffer, pH 2.0–6.0; 50 mM phosphate buffer, pH 6.0–8.0; 50 mM glycine sodium buffer, pH 8.0–11.0.

The effect of pH on PpPel9a was studied using PGA as a substrate. PpPel9a showed high activity over the pH range of pH 6 to pH 11, with optimum activity at pH 10 (Figure 3a). The reported PL9 pectate lyases showed different pH optima, such as Exo-PL from *E. chrysanthemi* EC16 (pH 7.5–8.0) [26], PelX from *E. chrysanthemi* 3937 (pH 8.5) [25], Pel-15H from Bacillus sp. strain KSM-P15 (pH 11.5) [24], and rPel9A from *C. stercorarium* (pH 7.0) [17]. The pH profile of PpPel9a was much broader than reported PL9 enzymes. So far, the pH stability of PL9 enzymes had not been reported. In this study, the pH stability profile of PpPel9a showed that the enzyme was stable over a wide pH range. After incubation between pH 5 and pH 11 for 24 h at 25 °C, residual activity remained greater than 75% (Figure 3b). The excellent alkali stability of PpPel9a makes the enzyme a good biocatalyst for pectin degradation.

**Figure 4:**
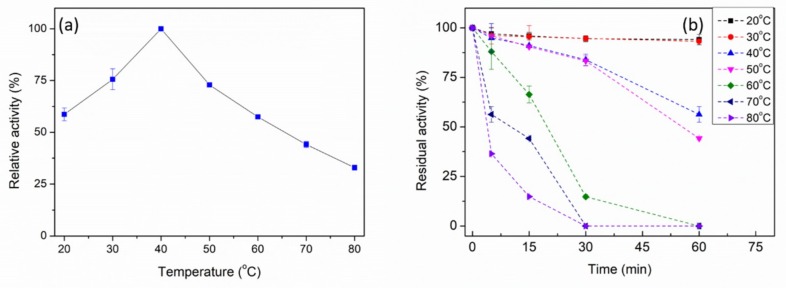
Effect of temperature on activity (**a**) and stability (**b**) of PpPel9a. The enzyme activity was detected by using 0.2% *w*/*v* PGA as a substrate.

The maximum activity of PpPel9a was observed at 40 °C (Figure 4a), which was lower than PL9 pectate lyases Pel-15H from *Bacillus* sp. strain KSM-P15 (55 °C) [24] and rPel9A from *C. stercorarium* F-9 (65 °C) [17]. A thermostability study showed that PpPel9a was stable below 50 °C, where more than 50% activity was maintained upon incubation for 60 min at 40 °C or 50 °C (Figure 4b). However, the activity decreased sharply when the incubation temperature reached 60 °C. Therefore, the thermostability of PpPel9a could be improved. 

The effect of metal ions and chemicals on the activity of PpPel9a was also evaluated, as shown in Table 1. 5 mM Tween-20 enhanced PpPel9a activity by 22.5%. Fe^2+^, Co^2+^, Tween-40, Tween-60, SDS, Tween-80, and Triton X-100 substantially decreased the activity of PpPel9a, and other metal ions examined did not significantly affect activity. Furthermore, different Tweens showed different effects on enzymatic activity. 

**Figure 5:**
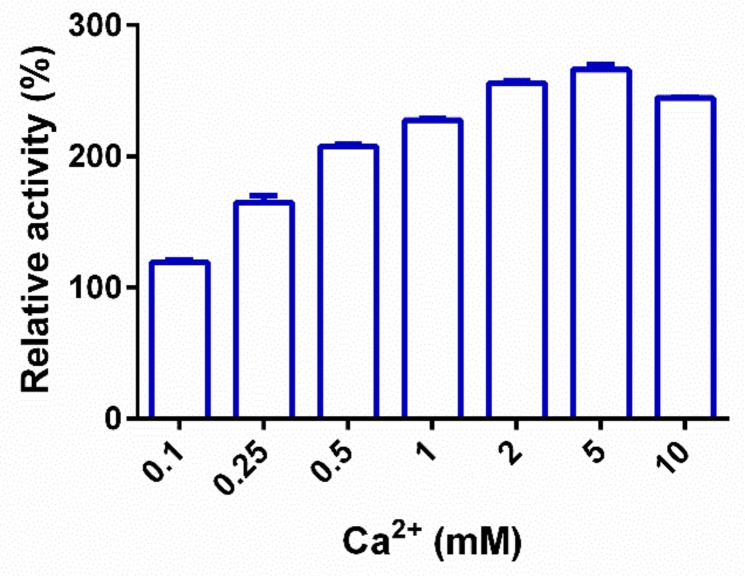
Effect of Ca^2+^ on the activity of PpPel9a. Values represent the mean ± SD (*n* = 3).

Generally, Ca^2+^ plays an important role in the hydrolytic action of pectate lyase. This ion acts by acidifying the C5 proton of the galacturonate binding to the +1 subsite of pectate lyase [27]. The enzymatic activities of PL9 enzymes are generally inhibited by treatment with EDTA. For instance, PelX from *E. chrysanthemi* 3937 and rPel9A from *C. stercorarium* F-9 are completely inhibited by 1 mM or 0.2 mM EDTA, respectively [17,25]. The activity of the PL10 enzyme PpPel10a is independent of Ca^2+^, although the enzymatic activity is significantly enhanced by its presence. Similar to PpPel10a, the activity of PpPel9a is not completely inhibited by EDTA, with over 76.5% of its enzymatic activity remaining. As shown in Figure 5, PpPel9a activity is enhanced by addition of Ca^2+^. In the presence of 5 mM Ca^2+^, PpPel19a activity is increased by 166.5%. Therefore, 5 mM Ca^2+^ was used in following experiments.

Michaelis–Menten parameters of PpPel9a on PGA and CP were determined. The *K*_m_ values of PpPel9a for PGA and CP were 0.18 and 0.32 g/L; *V*_max_ values were 298.5 and 107 μmol/min/mg, respectively, resulting in *k*_cat_/*K*_m_ values of 225.4 and 80.8 s^−1^, respectively.

### 2.4. Substrate Specificity Analysis of PpPel9a

Previously reported PL9 pectate lyases, such as Pel-15H from *Bacillus* sp. strain KSM-P15, PelX from *E. chrysanthemi* 3937, and rPel9A from *C. stercorarium* showed the highest catalytic activity for PGA, followed by pectins with low or medium methylation (<50%) [17,24,25]. Pectins with high degrees of methylation (≥50% methylation) were poor substrates for PL9 enzymes. However, the enzymatic activity of PL9 against RG pectins had yet to be reported. Here, the ability of PpPel9a to degrade various pectins, including RG-I type and HG-type pectins, were determined. As shown in Table 2, PpPel9a showed higher activity on HG-type pectins compared with RG-I type pectins. Using HG-type pectins as substrate, PpPel9a was more active on de-esterified HG-type pectins than on esterified HG-type pectins. The highest degradation activity was observed on de-esterified HG type pectin from citrus (CP-DeHG). Relative to the activity of PpPel9a on CP (100%), the activities of PpPel9a on CP-DeHG and CP-CeHG are 664% and 44%, respectively. Our results demonstrated that PL9 enzymes prefer low- or un-methylated HG pectins. 

**Figure 6:**
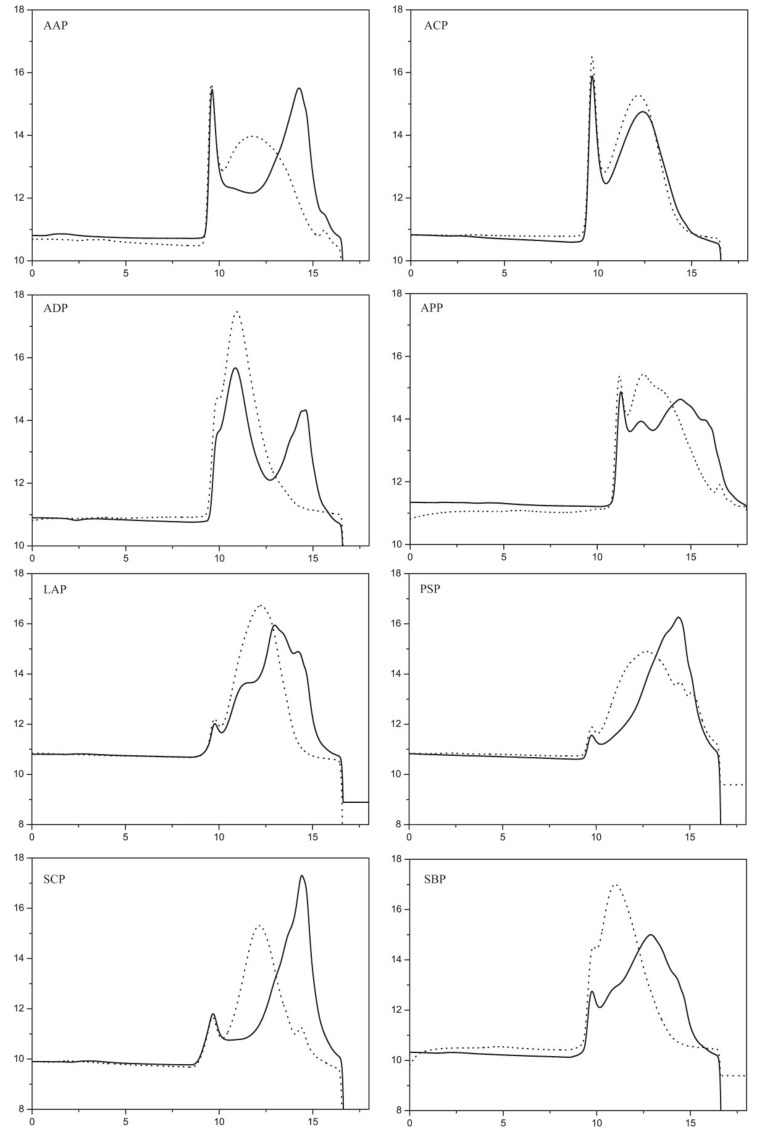
High-performance gel permeation chromatography (HPGPC) analysis of degradation of pectins from different plants by PpPel9a. Dash line represents the substrate and solid line represents the degradation product.

To explore the use of PpPel9a on the degradation of pectins, eight pectins from different plants were used as the substrates. The molecular weight distribution of the degradation products was detected by high-performance gel permeation chromatography (HPGPC). With the exception of ACP, PpPel9a degraded the pectins to varying degrees to produce low-molecular-weight fractions (Figure 6).

**Figure 7:**
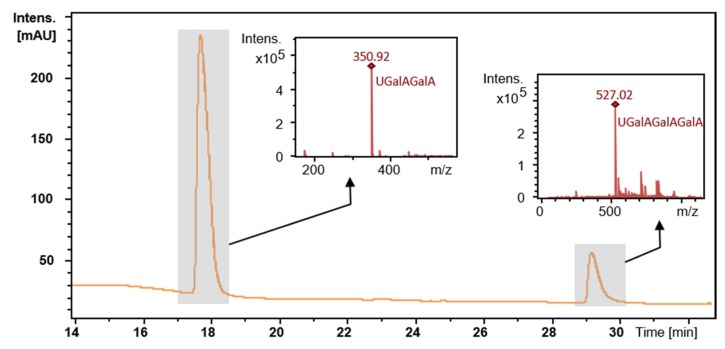
Analysis of the oligomers released from PGA by ultra-performance liquid chromatography-tandem mass spectrometer (UPLC-MS). The oligosaccharides separated by UPLC was further analyzed by ESI-MS analysis (small box).

Previously, pectate lyases were reported to exhibit catalytic activity via an endo- or exo-acting mode by β-elimination [27]. An exo-acting enzyme can produce 4,5-unsaturated GalA (uG1), while endo-acting enzymes tend to produce only unsaturated dimers or higher-degree polymerization unsaturated products. Two PL9 enzymes, PelX from *E. chrysanthemi* 3937 and Exo-PL from *E. chrysanthemi* EC16, have been reported as exo-acting pectate lyases, whereas Pel9A from *E. chrysanthemi* (1RU4) was reported to act as an endo-pectate lyase [25,26,27]. To assess the catalytic mechanism of PpPel9a, the oligomers released from CP by PpPel9a were analyzed by ultra-performance liquid chromatography - tandem mass spectrometer (UPLC-MS). As shown in Figure 7, no uG1 was observed upon degradation, which demonstrated that PpPel9a functions as an endo-acting enzyme. Electrospray ionisation mass spectrometry (ESI-MS) confirmed the UPLC results. Negative ESI-MS gave a strong peak at *m*/*z* 350.92 (unsaturated bigalacturonide, uG2), followed by *m*/*z* 527.02 (unsaturated trigalacturonide, uG3). These results indicate that citrus pectin was degraded by PpPel9a to produce a mixture of 4, 5-unsaturated oligo-galacturonic acid, confirming the trans-elimination endo-reaction mechanism of PpPel9a. The catalytic mechanism of PpPel9a was different from that of PpPel10a, which degraded citrus pectin to produce uG1 and 4,5-unsaturated oligomers, including uG2, uG3, uG4, and uG5. 

### 2.5. Degradation of Citrus Pectin and Characterization of the Main Degradation Product

**Figure 8:**
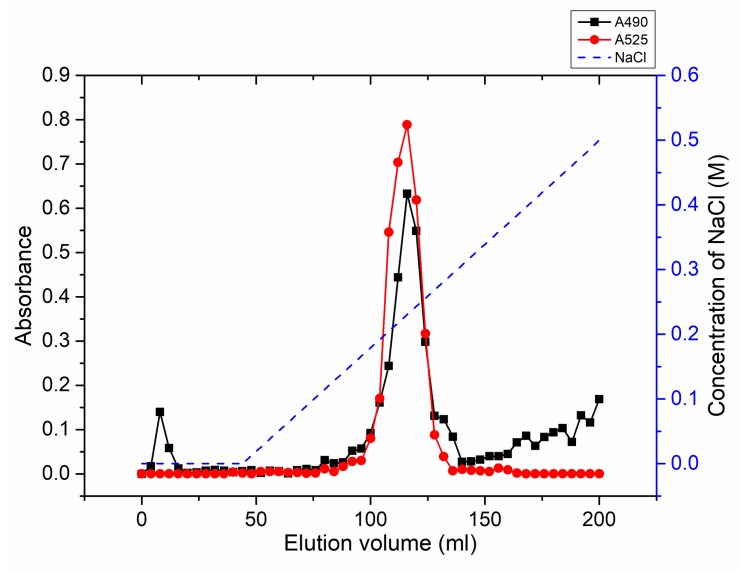
Elution profile of the degradation product of citrus pectin (CP) by DEAE sepharose fastflow column. The sugar content and uronic acid content were determined at 490 nm and 525 nm, respectively.

Previously, different methods including heat- and pH-modification were used to prepare modified citrus pectin. Jackson et al. showed that different treatment protocols of pectin can lead to different pectin activities, indicating that active molecules obtained from citrus pectin by different treatments are not the same [34]. Therefore, exploring new preparation techniques may be potentially useful for obtaining new active molecules. Here, citrus pectin was degraded by PpPel9a, and the degradation products were purified by using a DEAE sepharose fastflow column. As shown in Figure 8, a major peak was eluted by using 0.3 M NaCl and prolonging the degradation time or addition of more enzyme did not cause further degradation. This fraction is called MCP-0.3, and its molecular weight was determined to be ~24 kDa by HPGPC analysis (Figure S2). Monosaccharide composition analysis (Table 3) showed that after degradation, the content of GalA increased, while the content of Gal, Ara, and Glc decreased. The modified 2-Thiobarbituric acid (TBA) method indicated that the content of RGII domain increased in MCP-0.3 compared to that of CP (Figure S3). The Fourier Transform infrared spectroscopy (FT-IR) analysis showed that the degree of methyl esterification decreased from 65.5% in CP to 31.3% in MCP-0.3 (Figure S4), which indicated that the HG domain in CP was removed by PpPel9a degradation. Based on these results, we deduced that PpPel9a mainly cuts the HG domain in citrus pectin. Degradation experiments were repeated in triplicate, with similar results being obtained. The excellent reproducibility demonstrated that PpPel9a is suitable for citrus pectin degradation.

### 2.6. Growth Effect of MCP-0.3 on Lactobacillus Strains

Pectin fragments have been reported to exhibit good gastroprotective effects. For example, arabinogalactans from soybean significantly inhibit ethanol-induced gastric lesions in rats [35]. Two pectins ALR-a and ALR-b from rhizomes of *Atractylodes lancea* DC showed intestinal immune system modulating activity [36]. Oligosaccharides from pectin (POS) have been suggested as a new class of prebiotics, with anti-obesity, anti-toxic, anti-infection, anti-bacterial, and antioxidant properties [37]. Therefore, pectin may be a good source of prebiotic molecules. 

**Figure 9:**
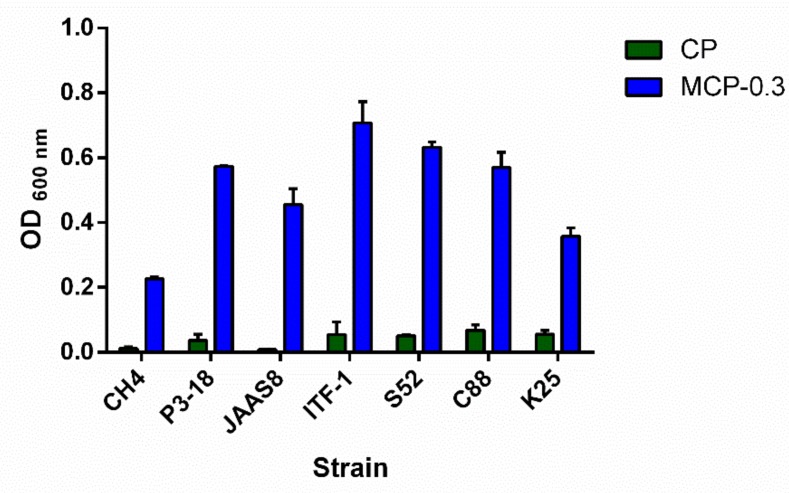
Effects of CP and MCP-0.3 on the growth of *L. plantarum* strains CH4, P3-18, S52, C88, K25, and *L. rhamnosus* CG strains JAAS8 and ITF-1.

The growth effect of freeze-dried MCP-0.3 was assayed in vitro with *Lactobacillus plantarum* strains CH4, P3-18, S52, C88, K25 and *L. rhamnosus* CG strains JAAS8 and ITF-1. As shown in Figure 9, compared with CP, MCP-0.3 at a concentration of 5 mg/mL significantly promotes the growth of *L. plantarum* and *L. rhamnosus* CG strains. The highest promotion effect was observed on *L. plantarum* S52 strain. Previously, the *B. subtilis* pectate lyase rePelB was reported to hydrolyze citrus pectin in a 50 L reactor. The low-molecular-weight citrus pectin (LCP) obtained significantly promoted the growth of probiotics *Bifidobacterium longum* [10]. In addition, the pectin oligosaccharide (POS) fractions obtained by controlled chemical degradation of citrus peel pectin were reported to promote the selective growth of probiotics *L. paracasei* LPC-37 and *B. bifidum* ATCC 29521 [38]. Here, we reported that the degradation product of citrus pectin by PpPel9a showed better prebiotic effects than citrus pectin, indicating that MCP-0.3 may be a good prebiotic agent. PpPel9a had the advantage of reproducible production of a low-molecular-weight fraction from citrus pectin, which was not the case with PpPel10a. Based on this, PpPel9a may be useful in preparing pectin fractions with prebiotic activity from pectin-rich agricultural wastes, such as citrus peel and apple pomace.

## 3. Materials and Methods

### 3.1. Strains and Reagents

*P. polymyxa* KF-1 was isolated and identified by our lab and preserved in China Center for Type Culture Collection (accession number CCTCC AB 2018146). It was used for supplying the PL9 pectate lyase gene. *E. coli* DH5α cells and pGM-Simple-T vector (TIANGEN, Beijing, China) were used for gene cloning. *E. coli* BL21 (DE3) cells and pET-28a (+) vector (Novagen, Temecula, California, USA) were selected for protein expression. *L. plantarum* strains CH4, P3-18, S52, C88, K25, and *L. rhamnosus* CG strains JAAS8 and ITF-1 were kindly provided by Dr Shengyu Li of the Institute of Agrofood Technology, Jilin Academy of Agricultural Sciences, China. Restriction enzymes and DNA polymerase were from TaKaRa Biotechnology Co., Ltd. (Dalian, China). DEAE-sepharose fastflow gel were purchased from GE Healthcare (USA). 

### 3.2. Substrates

Polygalacturonic acid (PGA) and citrus pectin (CP) were from Sigma-Aldrich (St. Louis, MO, USA). Pectins from *Panax* *ginseng* (PGP), red ginseng (RGP), *Anemarrhena asphodeloides* (AAP), *Asparagus cochinchinensis* (ACP), *Angelica dahurica* (ADP), *Angelica pubescens* (APP), *Leonurus Artemisia* (LAP), *Polygonatum sibiricum* (PSP), *Semen cassia* (SCP), and *Scutellaria baicalensis* (SBP) were isolated by hot water extraction followed by DEAE-cellulose fractionation according to the method previously reported by our lab [3,39]. The RG-I type pectins PGP-RGI from *P. ginseng*, and RGP-RGI from red ginseng, the HG type pectins PGP-HG from *P. ginseng*, RGP-HG from red ginseng, were prepared according to the method reported in Sun et al. [40]. The de-esterified HG type pectin CP-DeHG and the completely esterified HG type pectin CP-CeHG were prepared from citrus pectin according to the method reported in Sun et al. [40].

### 3.3. Analytical Methods

The unsaturated oligomers released from CP by PpPel9a were analyzed by UPLC-MS. The UPLC-MS was performed using a Waters Acquity UPLC system with tandem UV (232 nm) and ESI-MS detector. The oligosaccharides were first separated by Acquity UPLC BEH Amide column (1.7 μm, 2.1 mm × 150 mm) eluted at a flow rate of 300 μL/min and a column oven temperature of 35 °C. The mobile phase consisted of three mobile phase lines, (A) 20:80 (*v*/*v*) ACN/water, (B) 80:20 (*v*/*v*) ACN/water, and (C) 200 mM ammonium formate/50 mM formic acid buffer (pH 3.0). The elution program was as follows: 0–30 min, 95%−75% B; 30–31 min, 75%−60% B; 31–40 min, isocratic with 60% B; 40–41 min, 60%−95% B; 41–50 min, isocratic with 95% B; 5% buffer (C) was constantly added throughout the elution. ESI-MS analysis was performed with a Bruker Daltonics Amazon mass spectrometer equipped with an electrospray source and an ion trap mass analyzer (Bremen, Germany). The spectrometer was operated in the negative ion mode (capillary voltage, 4 kV; end plate off set: −300 V; temperature, 200 °C; nebulizer gas: 2 bar and dry gas, 6 mL/min). Monosaccharide composition was analyzed by high performance anion exchange chromatography (HPAEC) method using a CarboPac PA20 column (3 × 150 mm) assembled to a Dionex ICS-5000 Plus ion chromatographic system [41]. Homogeneity and molecular weight of polysaccharides were estimated by HPGPC with a TSK-gel G-3000PWXL column (7.8 × 300 mm, TOSOH, Japan) coupled to a Shimadzu high performance liquid chromatography (HPLC) system. The column was pre-calibrated using standard dextrans (1, 5, 12, 25, and 50 kDa) [42]. The degree of esterification was determined by FT-IR. The degree of methyl esterification = the area of methyl-esterified carboxyl group/the area of total carboxyl group × 100% [43]. Kdo and Dha were colorimetrically determined using the modified TBA method to check the presence of RG-II domain in polysaccharides [44]. 

### 3.4. Analysis of Pectate Lyase Gene

The ORF of the pectate lyase was analyzed by NCBI ORF finder [45]. The conserved domain was explored by Pfam [46]. The molecular weight (Mw) and isoelectric point (pI) were calculated by Compute pI/Mw too [47]. The signal peptide was predicted by SignalP 5.0 Server [48]. Multiple amino acid sequences alignments were performed by Clustal Omega and exhibited by ESPript 3.0 web server [49,50]. The structure of pectate lyase PpPel9a was predicted by SWISS-MODEL with the pectate lyase from *E. chrysanthemi* (PDB accession number: 1RU4) as the template [27]. The model was exhibited by Pymol software [51].

### 3.5. Cloning of PL9 Pectate Lyase and Its Expression

The mature pectate lyase encoding gene without the signal peptide sequence was obtained from the genomic DNA of *P. polymyxa* KF-1 by PCR using following primers: 5′-CGCATATGGATGTTCCATCTAACAACCTC-3′ and 5′-CGGGATCCTTACCGTGCCCCTATATTTCCGC-3′. The underlines indicate the *Nde*I and *Bam*HI restriction sites, respectively. The PCR amplification was performed by PrimeSTAR^®^ HS DNA polymerase with the following program: Pre-denaturation at 98 °C for 10 s; 30 cycles of 98 °C for 10 s, 55 °C for 15 s, and 72 °C for 1.5 min; final extension at 72 °C for 10 min. The PCR product was ligased with pGM-Simple-T vector, transformed into *E. coli* DH5α competent cells, and plated onto Luria–Bertani (LB) agar plates supplemented with 100 μg/mL ampicillin according to the manufacturer’s instructions. Recombinant plasmid was sequenced with T7 (5’-ACATCCACTTTGCCTTTCTC-3’) and SP6 (5’-ATTTAGGTGACACTATAG-3’) as sequencing primers. The recombinant plasmid with correct insert DNA was double digested by *Nde*I and *Bam*HI, and ligased into pET-28a (+) vector digested with the same restriction enzymes. The recombinant plasmid was transformed into *E. coli* BL21 (DE3) cells and plated onto LB agar plates supplemented with 30 μg/mL kanamycin.

The positive colony was incubated in 500 mL of LB broth at 37 °C for 3 h with shaking at 200 rpm. Then, the isopropyl β-d-thiogalactoside (IPTG) was added to a final concentration of 0.5 mM. After induced at 25 °C for 20 h, the cells were centrifuged at 5000 rpm and 4 °C for 10 min. Then, the cells were sonicated on ice and centrifuged at 12,000× *g* and 4 °C for 10 min to remove cell debris. The cell lysate was loaded onto a Ni^2+^–NTA agarose chromatography (column volume 5 mL), which was pre-equilibrated with equilibration buffer (20 mM Tris–HCl, 100 mM NaCl, 20 mM imidazole, pH 8.0). Binding proteins were eluted with a linear gradient of imidazole (20–200 mM) in 20 mM Tris–HCl, 100 mM NaCl, pH 8.0. Fractions with enzymatic activity were pooled and dialyzed against 50 mM Tris–HCl buffer (pH 9.0). The cell lysate and purified enzyme were analyzed by SDS-PAGE using 10% separating gel and 3.9% stacking gel [52]. The protein concentration of the purified enzyme was determined using BCA Protein Assay Kit (Beyotime, Beijing, China).

### 3.6. Pectate Lyase Activity Assay and Substrate Specificity Analysis of PpPel9a

During expression and purification, the enzymatic activity of PpPel9a was measured by determining the formed unsaturated bonds at 235 nm with an UV-5100H spectrophotometer (METASH, Shanghai, China) with soluble PGA as the substrate [19]. The reaction mixture of 200 μL consists of 0.2% (*w*/*v*) PGA, 5 mM CaCl_2_, and 2 μg of purified enzyme was incubated in 50 mM Tris-HCl buffer (pH 9.0). The reaction mixture was incubated at 40 °C for 30 min and the unsaturated bonds produced by enzymatic degradation were determined at 235 nm. One unit of pectate lyase activity was defined as the enzyme needed to produce 1 μmol of unsaturated bonds per min. The molar extinction coefficient was 4600 M^−1^cm^−1^. 

The substrate specificity of recombinant PpPel9a was determined using different types of pectins, including HG, RG-I, de-esterified HG, and completely-esterified HG, under standard reaction conditions (pH 10.0, 40 °C, 5 mM CaCl_2_, 0.2% *w*/*v* substrate). The unsaturated bonds released from the substrates were recorded at 235 nm. The molecular weights of the degradation products were analyzed by HPGPC method. 

The kinetic parameters of PpPel9a on PGA and CP were determined by the A235 method. Purified recombinant PpPel9a was incubated with PGA or CP at different concentrations (0.1–5 mg/mL) at 40 °C in 50 mM glycine–NaOH buffer (pH 10.0) for 10 min. Then, the unsaturated bonds formed were measured at 235 nm. The *K*_m_ and *k*_cat_ values were calculated by GraphPad Prism5.0 software using non-linear regression. All data are expressed as the means of triplicate measurements.

### 3.7. Effect of pH, Temperature, and Metal Ions on the Activity and Stability of PpPel9a

The optimal pH of PpPel9a was determined at 40 °C for 30 min in 50 mM NaAC buffer (pH 2.0–6.0), 50 mM phosphate buffer (pH 6.0–8.0), and 50 mM glycine–NaOH buffer (pH 8.0–11.0). The temperature optimum of PpPel9a was measured at 20–80 °C for 30 min in 50 mM glycine–NaOH (pH 10.0). The pH stability of PpPel9a was assayed by measuring its residual activity after incubating the purified enzyme in 50 mM NaAC buffer (pH 2.0–6.0), 50 mM phosphate buffer (pH 6.0–8.0), and 50 mM glycine–NaOH buffer (pH 8.0–11.0) at 25 °C for 24 h. The thermal stability of PpPel9a was determined by measuring the residual enzyme activity after incubating the purified enzyme at 20–80 °C in 50 mM glycine–NaOH buffer (pH 10.0) for up to 1 h. In enzymatic characterization, soluble PGA was used as the substrate with the concentration of 0.2% (*w*/*v*), 5 mM CaCl_2_ was added during enzymatic reaction. Formed unsaturated products were recorded at 235 nm. All data are expressed as the averages of triplicate measurements (± SD).

The effects of metal ions and chemical reagents on PpPel9a activity were detected by incubating the purified enzyme in 5 mM of metal ions and reagents at 25 °C for 24 h, then the residual activity was measured under the standard assay condition with 0.2% (*w*/*v*) PGA as the substrate. The enzymatic activity without adding any metal ions or chemical reagents was marked as 100%. The effect of Ca^2+^ at different concentrations (0.1–10 mM) on enzyme activity was determined in 50 mM glycine–NaOH buffers (pH 10.0) with 0.2% PGA (*w*/*v*) as the substrate. Formed unsaturated products were recorded at 235 nm. The enzymatic activity without adding CaCl_2_ was marked as 100%.

### 3.8. Degradation of CP by PpPel9a and the Preparation of Degradation Product

First, 2.5 g of CP was incubated with 50 mg of purified recombinant PpPel9a in 50 mM Tris-HCl buffer (pH 8.0). The enzyamtic degradation lasted for 48 h at 40 °C. Then, the reaction mixture was boiled for 10 min and centrifuged at 10,000× *g* for 10 min to remove the protein. Then, the degradation product was freeze-dried and purified by DEAE-sepharose fastflow column. The column (bed volume 25 mL) was eluted by 2 column volume of water, followed by 6 column volume of linear gradient of 0–0.5 M NaCl. The eluate was collected at 4 mL/tube and detected for the distribution of total sugar by the phenol-sulphuric acid method, and uronic acid by the m-hydroxydiphenyl method [53,54]. The main fraction peak eluted by 0.3 M NaCl was combined, dialyzed against distilled water, and freeze-dried. The fraction was named MCP-0.3, and its monosaccharide composition, molecular weight, RG-II content, and degree of esterification were analyzed by HPAEC, HPGPC, modified TBA method, and FT-IR, respectively.

### 3.9. Prebiotic Effect of CP and Its Degradation Product MCP-0.3 on Lactobacillus Strains

The prebiotic effects of CP and MCP-0.3 on Lactobacillus strains were studied as described in Zheng et al. [41]. Briefly, Lactobacillus strains, including *L. rhamnosus* strains JAAS8 and ITF-1, and *L. plantarum* strains CH4, P3-18, S52, C88, and K25, were pre-cultured in De Man Rogosa Sharpe (MRS) liquid at 37 °C for 16 h. Then, the cells were inoculated with an inoculum size of 2% in 100 μL modified MRS media containing 5 mg/mL CP or MCP-0.3 as the sole carbon source. Then, the cells were collected by centrifugation after cultured at 37 °C for 16 h and washed twice with PBS buffer. The cell pellet was re-suspended in 200 μL PBS buffer, and the OD _600 nm_ was detected by the Tecan Infinite F50 microplate reader. All data are shown as the averages of triplicate measurements (± SD).

## 4. Conclusions

In the present study, the PL9 pectate lyase PpPel9a was cloned from *P. polymyxa* KF-1 and was used to degrade pectins from different plants. Recombinant PpPel9a was used to degrade citrus pectin, reproducibly yielding the fraction MCP-0.3 that exhibited good prebiotic effects in vitro. This enzyme may be a valuable candidate for preparation of low-molecular-weight pectin fractions.

## Figures and Tables

**Table 1:** Effect of various metal ions and chemicals on the activity of PpPel9a.

Metal Ions or Chemicals (5 mM)	Relative Activity (%) ^1^
NaCl	86.7 ± 3.3
KCl	79.0 ± 0.6
CaCl_2_	266.5 ± 7.0
MgCl_2_	94.5 ± 4.6
FeSO_4_	54.5 ± 0.7
EDTA	63.1 ± 0.5
ZnCl_2_	88.0 ± 1.6
AlCl_3_	106.1 ± 6.1
CoCl_2_	51.4 ± 2.4
SDS	37.7 ± 0.2
Tween-20	122.5 ± 0.6
Tween-40	51.4 ± 2.1
Tween-60	40.0 ± 0.6
Tween-80	2.0 ± 0.1
TritonX-100	4.3 ± 0.3

^1^ The activity was determined with 0.2% *w*/*v* PGA as a substrate.

**Table 2:** Degradation of various pectins by PpPel9a.

Type of Pectin	Substrate ^1^	Specific Activity (U/mg)
Pectin	PGP	48.1 ± 4.9
	RGP	59.1 ± 0.1
	CP	107.0 ± 0.2
RG-I	PGP-RGI	2.1 ± 0.9
	RGP-RGI	21.0 ± 0.3
HG	PGP-HG	106.8 ± 0.6
	RGP-HG	68.9 ± 0.5
Completely esterified HG	CP-CeHG	47.1 ± 0.1
De-esterified HG	CP-DeHG	710.7 ± 3.3

^1^ CP: citrus pectin; PGP: pectin from *Panax ginseng*; RGP: pectin from red ginseng.

**Table 3:** Monosaccharide composition of CP and its degradation product MCP-0.3.

	Monosaccharide (%)								
	Man	GlcA	Rha	GalA	Glc	Gal	Xyl	Ara	Fuc
CP	1.20	0.57	3.62	62.18	4.12	13.73	- ^1^	14.42	0.16
MCP-0.3	1.12	1.66	3.68	86.97	1.02	2.06	-	2.49	1.00

^1^ Not detected.

## Supplementary Materials

Supplementary materials can be found at https://www.mdpi.com/1422-0067/20/12/3060/s1.

## References

[1] Christiaens S., Van Buggenhout S., Houben K., Jamsazzadeh Kermani Z., Moelants K.R.N., Ngouemazong E.D., Van Loey A., Hendrickx M.E.G. (2016). Process-Structure-Function Relations of Pectin in Food. Crit. Rev. Food Sci. Nutr..

[2] Chan S.Y., Choo W.S., Young D.J., Loh X.J. (2017). Pectin as a rheology modifier: Origin, structure, commercial production and rheology. Carbohydr. Polym..

[3] Zhang X., Li S., Sun L., Ji L., Zhu J., Fan Y., Tai G., Zhou Y. (2012). Further analysis of the structure and immunological activity of an RG-I type pectin from *Panax ginseng*. Carbohydr. Polym..

[4] Minzanova S.T., Mironov V.F., Arkhipova D.M., Khabibullina A. V., Mironova L.G., Zakirova Y.M., Milyukov V.A. (2018). Biological Activity and Pharmacological Application of Pectic Polysaccharides: A Review. Polymers (Basel)..

[5] Liu Y., Dong M., Yang Z., Pan S. (2016). Anti-diabetic effect of citrus pectin in diabetic rats and potential mechanism via PI3K/Akt signaling pathway. Int. J. Biol. Macromol..

[6] Chen J., Ye F., Zhou Y., Zhao G. (2018). Thiolated citrus low-methoxyl pectin: Synthesis, characterization and rheological and oxidation-responsive gelling properties. Carbohydr. Polym..

[7] Ruvolo P.P. (2016). Galectin 3 as a guardian of the tumor microenvironment. Biochim. Biophys. Acta Mol. Cell Res..

[8] Wang S., Li P., Lu S.-M., Ling Z.-Q. (2016). Chemoprevention of Low-Molecular-Weight Citrus Pectin (LCP) in Gastrointestinal Cancer Cells. Int. J. Biol. Sci..

[9] do Prado S.B.R., Shiga T.M., Harazono Y., Hogan V.A., Raz A., Carpita N.C., Fabi J.P. (2019). Migration and proliferation of cancer cells in culture are differentially affected by molecular size of modified citrus pectin. Carbohydr. Polym..

[10] Liu M., Huo W., Dai X., Dang Y. (2018). Preparation of low-molecular-weight citrus pectin by recombinant *Bacillus subtilis* pectate lyase and promotion of growth of *Bifidobacterium longum*. Catal. Commun..

[11] Chen J., Liu W., Liu C.-M., Li T., Liang R.-H., Luo S.-J. (2015). Pectin modifications: A review. Crit. Rev. Food Sci. Nutr..

[12] Patidar M.K., Nighojkar S., Kumar A., Nighojkar A. (2018). Pectinolytic enzymes-solid state fermentation, assay methods and applications in fruit juice industries: A review. 3 Biotech.

[13] Jolie R.P., Duvetter T., Van Loey A.M., Hendrickx M.E. (2010). Pectin methylesterase and its proteinaceous inhibitor: A review. Carbohydr. Res..

[14] Herron S.R., Benen J.A., Scavetta R.D., Visser J., Jurnak F. (2000). Structure and function of pectic enzymes: Virulence factors of plant pathogens. Proc. Natl. Acad. Sci. USA.

[15] Marin-Rodriguez M.C., Orchard J., Seymour G.B. (2002). Pectate lyases, cell wall degradation and fruit softening. J. Exp. Bot..

[16] Hugouvieux-Cotte-Pattat N., Condemine G., Shevchik V.E. (2014). Bacterial pectate lyases, structural and functional diversity. Environ. Microbiol. Rep..

[17] Hla S.S., Kurokawa J., Suryani, Kimura T., Ohmiya K., Sakka K. (2005). A novel thermophilic pectate lyase containing two catalytic modules of *Clostridium stercorarium*. Biosci. Biotechnol. Biochem..

[18] Lombard V., Golaconda Ramulu H., Drula E., Coutinho P.M., Henrissat B. (2014). The carbohydrate-active enzymes database (CAZy) in 2013. Nucleic Acids Res..

[19] Zhou C., Xue Y., Ma Y. (2017). Cloning, evaluation, and high-level expression of a thermo-alkaline pectate lyase from alkaliphilic *Bacillus clausii* with potential in ramie degumming. Appl. Microbiol. Biotechnol..

[20] Zhang C., Yao J., Zhou C., Mao L., Zhang G., Ma Y. (2013). The alkaline pectate lyase PEL168 of *Bacillus subtilis* heterologously expressed in *Pichia pastoris* is more stable and efficient for degumming ramie fiber. BMC Biotechnol..

[21] van Rensburg P., Strauss M.L.A., Lambrechts M.G., Cordero Otero R.R., Pretorius I.S. (2007). The heterologous expression of polysaccharidase-encoding genes with oenological relevance in *Saccharomyces cerevisiae*. J. Appl. Microbiol..

[22] Willems J.L., Low N.H. (2016). Oligosaccharide formation during commercial pear juice processing. Food Chem..

[23] Zhao Y., Yuan Y., Zhang X., Li Y., Li Q., Zhou Y., Gao J. (2018). Screening of a Novel Polysaccharide Lyase Family 10 Pectate Lyase from *Paenibacillus polymyxa* KF-1: Cloning, Expression and Characterization. Molecules.

[24] Ogawa A., Sawada K., Saito K., Hakamada Y., Sumitomo N., Hatada Y., Kobayashi T., Ito S. (2000). A new high-alkaline and high-molecular-weight pectate lyase from a *Bacillus* isolate: Enzymatic properties and cloning of the gene for the enzyme. Biosci. Biotechnol. Biochem..

[25] Shevchik V.E., Kester H.C., Benen J.A., Visser J., Robert-Baudouy J., Hugouvieux-Cotte-Pattat N. (1999). Characterization of the exopolygalacturonate lyase PelX of *Erwinia chrysanthemi* 3937. J. Bacteriol..

[26] Brooks A.D., He S.Y., Gold S., Keen N.T., Collmer A., Hutcheson S.W. (1990). Molecular cloning of the structural gene for exopolygalacturonate lyase from *Erwinia chrysanthemi* EC16 and characterization of the enzyme product. J. Bacteriol..

[27] Jenkins J., Shevchik V.E., Hugouvieux-Cotte-Pattat N., Pickersgill R.W. (2004). The crystal structure of pectate lyase Pel9A from *Erwinia chrysanthemi*. J. Biol. Chem..

[28] Luis A.S., Briggs J., Zhang X., Farnell B., Ndeh D., Labourel A., Basle A., Cartmell A., Terrapon N., Stott K. (2018). Dietary pectic glycans are degraded by coordinated enzyme pathways in human colonic Bacteroides. Nat. Microbiol..

[29] Park S.R., Kim M.K., Kim J.O., Bae D.W., Cho S.J., Cho Y.U., Yun H.D. (2000). Characterization of *Erwinia chrysanthemi* PY35 cel and pel gene existing in tandem and rapid identification of their gene products. Biochem. Biophys. Res. Commun..

[30] Zhou C., Ye J., Xue Y., Ma Y. (2015). Directed Evolution and Structural Analysis of Alkaline Pectate Lyase from the Alkaliphilic Bacterium *Bacillus* sp. Strain N16-5 To Improve Its Thermostability for Efficient Ramie Degumming. Appl. Environ. Microbiol..

[31] Hao M., Yuan X., Cheng H., Xue H., Zhang T., Zhou Y., Tai G. (2013). Comparative studies on the anti-tumor activities of high temperature- and pH-modified citrus pectins. Food Funct..

[32] Pickersgill R., Jenkins J., Harris G., Nasser W., Robert-Baudouy J. (1994). The structure of *Bacillus subtilis* pectate lyase in complex with calcium. Nat. Struct. Biol..

[33] Liu Y., Chen G., Wang J., Hao Y., Li M., Li Y., Hu B., Lu F. (2012). Efficient expression of an alkaline pectate lyase gene from *Bacillus subtilis* and the characterization of the recombinant protein. Biotechnol. Lett..

[34] Jackson C.L., Dreaden T.M., Theobald L.K., Tran N.M., Beal T.L., Eid M., Gao M.Y., Shirley R.B., Stoffel M.T., Kumar M.V. (2007). Pectin induces apoptosis in human prostate cancer cells: Correlation of apoptotic function with pectin structure. Glycobiology.

[35] Cipriani T.R., Mellinger C.G., Bertolini M.L.C., Baggio C.H., Freitas C.S., Marques M.C.A., Gorin P.A.J., Sassaki G.L., Iacomini M. (2009). Gastroprotective effect of a type I arabinogalactan from soybean meal. Food Chem..

[36] Yu K.-W., Kiyohara H., Matsumoto T., Yang H.-C., Yamada H. (2001). Characterization of pectic polysaccharides having intestinal immune system modulating activity from rhizomes of *Atractylodes lancea* DC. Carbohydr. Polym..

[37] Di R., Vakkalanka M.S., Onumpai C., Chau H.K., White A., Rastall R.A., Yam K., Hotchkiss A.T.J. (2017). Pectic oligosaccharide structure-function relationships: Prebiotics, inhibitors of *Escherichia coli* O157:H7 adhesion and reduction of Shiga toxin cytotoxicity in HT29 cells. Food Chem..

[38] Zhang S., Hu H., Wang L., Liu F., Pan S. (2018). Preparation and prebiotic potential of pectin oligosaccharides obtained from citrus peel pectin. Food Chem..

[39] Zhang X., Yu L., Bi H., Li X., Ni W., Han H., Li N., Wang B., Zhou Y., Tai G. (2009). Total fractionation and characterization of the water-soluble polysaccharides isolated from *Panax ginseng* C. A. Meyer. Carbohydr. Polym..

[40] Sun L., Ropartz D., Cui L., Shi H., Ralet M.-C., Zhou Y. (2019). Structural characterization of rhamnogalacturonan domains from *Panax ginseng* C. A. Meyer. Carbohydr. Polym..

[41] Zheng Y., Li L., Feng Z., Wang H., Mayo K.H., Zhou Y., Tai G. (2018). Preparation of individual galactan oligomers, their prebiotic effects, and use in estimating galactan chain length in pectin-derived polysaccharides. Carbohydr. Polym..

[42] Wu D., Cui L., Yang G., Ning X., Sun L., Zhou Y. (2018). Preparing rhamnogalacturonan II domains from seven plant pectins using *Penicillium oxalicum* degradation and their structural comparison. Carbohydr. Polym..

[43] Peng X., Yang G., Fan X., Bai Y., Ren X., Zhou Y. (2016). Controlled methyl-esterification of pectin catalyzed by cation exchange resin. Carbohydr. Polym..

[44] York W.S., Darvill A.G., McNeil M., Albersheim P. (1985). 3-deoxy-d-manno-2-octulosonic acid (KDO) is a component of rhamnogalacturonan II, a pectic polysaccharide in the primary cell walls of plants. Carbohydr. Res..

[45] Sayers E.W., Barrett T., Benson D.A., Bolton E., Bryant S.H., Canese K., Chetvernin V., Church D.M., DiCuccio M., Federhen S. (2011). Database resources of the National Center for Biotechnology Information. Nucleic Acids Res..

[46] Finn R.D., Bateman A., Clements J., Coggill P., Eberhardt R.Y., Eddy S.R., Heger A., Hetherington K., Holm L., Mistry J. (2014). Pfam: The protein families database. Nucleic Acids Res..

[47] Wilkins M.R., Gasteiger E., Bairoch A., Sanchez J.C., Williams K.L., Appel R.D., Hochstrasser D.F. (1999). Protein identification and analysis tools in the ExPASy server. Methods Mol. Biol..

[48] Almagro Armenteros J.J., Tsirigos K.D., Sonderby C.K., Petersen T.N., Winther O., Brunak S., von Heijne G., Nielsen H. (2019). SignalP 5.0 improves signal peptide predictions using deep neural networks. Nat. Biotechnol..

[49] Sievers F., Higgins D.G. (2014). Clustal omega. Curr. Protoc. Bioinforma..

[50] Gouet P., Robert X., Courcelle E. (2003). ESPript/ENDscript: Extracting and rendering sequence and 3D information from atomic structures of proteins. Nucleic Acids Res..

[51] Mooers B.H.M. (2016). Simplifying and enhancing the use of PyMOL with horizontal scripts. Protein Sci..

[52] Schagger H. (2006). Tricine-SDS-PAGE. Nat. Protoc..

[53] DuBois M., Gilles K.A., Hamilton J.K., Rebers P.A., Smith F. (1956). Colorimetric Method for Determination of Sugars and Related Substances. Anal. Chem..

[54] Blumenkrantz N., Asboe-Hansen G. (1973). New method for quantitative determination of uronic acids. Anal. Biochem..

