# Supplementary material for: A Novel PL9 Pectate Lyase from Paenibacillus polymyxa KF-1: Cloning, Expression, and Its Application in Pectin Degradation
Source: Int J Mol Sci. 2019 Jun 22;20(12):3060. doi: 10.3390/ijms20123060 (PMC6627557; doi:10.3390/ijms20123060)
Supplement: Supplementary file 1 [file ijms-20-03060-s001.pdf]

A novel PL9 pectate lyase from *Paenibacillus polymyxa* KF-1: cloning, expression and its application in pectin degradation

Ye Yuan[1], Xinyu Zhang[2], Yan Zhao[2], Han Zhang[1], Yifa Zhou[1], and Juan Gao[1, 2, *]

[1] School of Life Sciences, Northeast Normal University, Changchun 130024, PR China; yuany268@nenu.edu.cn (Y.Y.); zhangh800@nenu.edu.cn (H.Z.); zhouyf383@nenu.edu.cn (Y.-F.Z)

[2] School of Biological Science and Technology, University of Jinan, Jinan 250022, PR China; zhangxinyu950512@163.com (X.-Y.Z); zhaoyan_1994@126.com (Y.Z.); bio_gaoj@ujn.edu.cn (J.G.)

* Correspondence: bio_gaoj@ujn.edu.cn (J.G.); Tel.: +86-531-89736825

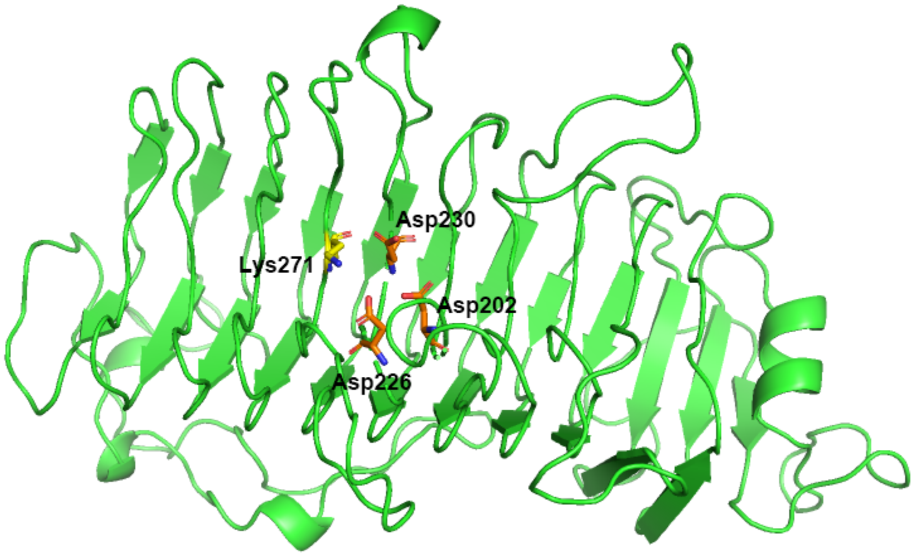

Figure S1. The three-dimensional structure model of PpPel9a predicted by SWISS-MODEL with Pel9A from *E. chrysanthemi* (1RU4) as the template. Amino acids 23-416 of PpPel9a were covered by the model. Asp202, Asp226 and Asp230, labeled in orange, are predicted to be putative catalytic residues. Lys 217 labeled in yellow is predicted to be putative catalytic base.

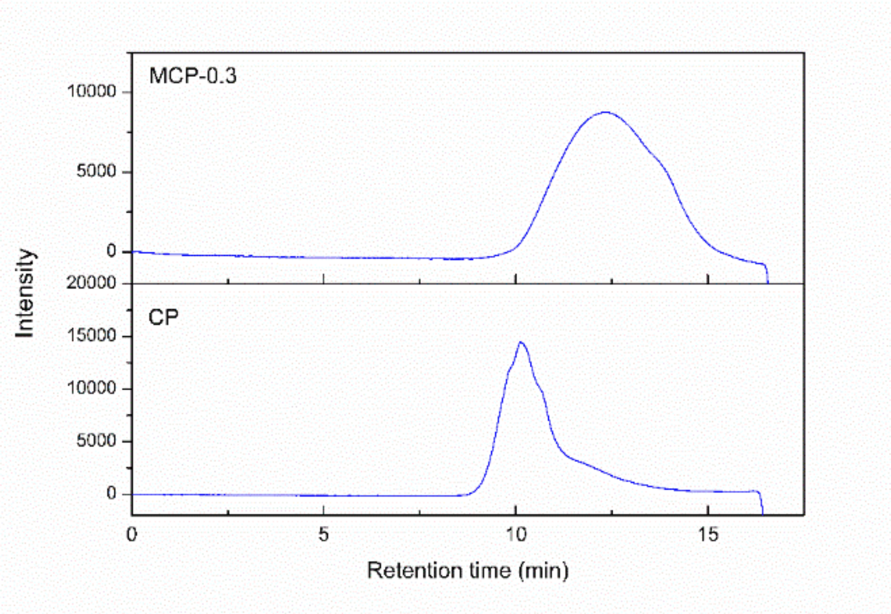

Figure S2. HPGPC analysis of CP and its degradation product MCP-0.3 by PpPel9a.

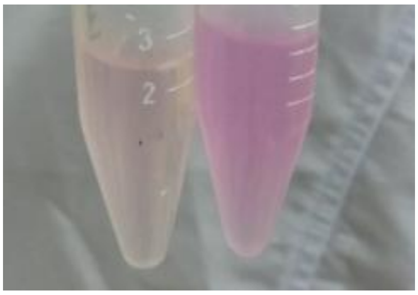

Figure S3. Modified TBA method to determine the RG-II domain in CP (left) and MCP-0.3 (right).

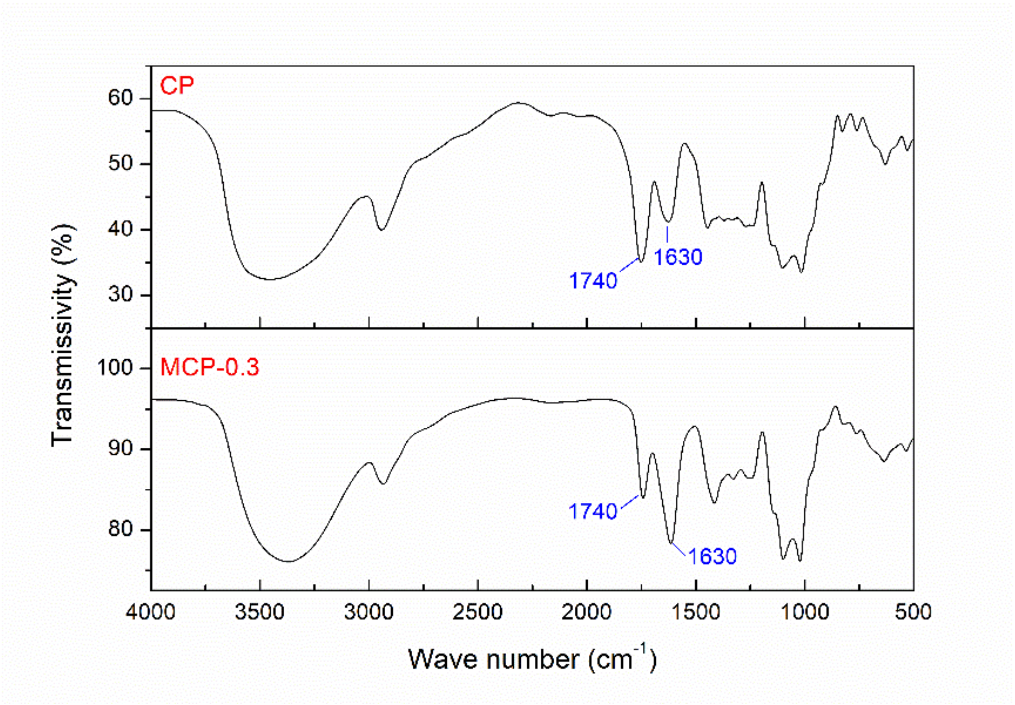

Figure S4. FT-IR analysis of CP (a) and its degradation product MCP-0.3 by PpPel9a (b). The degree of methyl esterification = the area of methyl-esterified carboxyl group/the area of total carboxyl group × 100%. Specific bands at 1740 and 1630 cm^{-1} corresponded to the absorption of the esterified carbonyl groups and carboxylic ions, respectively.
